# 

**Published:** 1902-05-17

**Authors:** 


### NOTICE TO CORRESPONDENTS.

All MSS., Letters, Books for Review, and other matters intended for the Editor should be addressed The Editor, "The Hospital," The Hospital Building, 28 & 29 Southampton Street, Strand, W.C.
The Editor cannot undertake to return rejected MS., even when accompanied by stamped directed envelope.

**Notice to Advertisers and Subscribers.**
All Advertisements, Orders for Copies of The Hospital, and Business Communications should be addressed to **THE MANAGER, (Not to the Editor)**, The Hospital, 28 & 29 Southampton Street, Strand, London, W.C.
The Cost of Subscription, post free, is as follows.—Per week, 2½d.; per quarter, 2s. 8d.; per half-year, 5s. 3d.; per annum, 10s. 6d. Foreign:—Per annum, 15s. 2d., payable, in all cases, in advance.

**NOTICE.—Vol. XXXI. of "The Hospital" (October 1901, to March 1902), in handsome cloth binding is now on sale at the office of this Journal, price, 7s. 6d. Cases for binding the Numbers contained in this Volume 1s. 6d. Index to Vol. XXXI., 2½d., post free.**
**The Monthly Part for June will be ready on June 2nd. Price 6d., by post 8d.**

### BOOKS RECEIVED.

J. B. Lippincott and Co.

"The Technics of Nephropexy." By George M. Edebohls, M.D.
"Photographic Atlas of Diseases of the Skin." By George Henry Fox, A.M., M.D.

Florence White.

"Sick Room Cookery."

John Wright and Co.

"An Introduction to Dermatology." By Norman Walker, M.D.

### SCRAPS AND GLEANINGS.

By a unanimously carried resolution of the annual meeting of the Cardiff, Penarth, and Barry Coal Trimmers' Mutual Friendly Society it was decided that the coal trimmers of Cardiff should organise a regular subscription throughout their union, to which every man should make a small weekly contribution, with the object of providing a fund to put the Cardiff Infirmary on a sound financial basis.

During 1901 the number of in-patients received into the Kent County Ophthalmic Hospital was 250 against 303 in 1900. The number of out-patients shows a slight increase. The ordinary income for the year was £1,615, and the ordinary expenditure £1,567, as against £1,679 and £1,690 respectively in 1900. The renewal of the water service pipes throughout the hospital, which was found necessary during the year, was paid for out of capital, as the annual income was insufficient to meet this expenditure.

### THE STUDENT OF MEDICINE.

### APPOINTMENTS.

**C. R. H. Ball**, M.R.C.S., L.R.C.P.Lond., appointed Assistant House Physician to the Metropolitan Hospital. **Comyns Berkeley**, M.B., B.C.Cantab., M.R.C.P.Lond., appointed Lecturer on Practical Midwifery at the Middlesex Hospital. **Thomas Borthwick**, M.D.Edin., appointed Honorary Bacteriologist to the Adelaide Hospital, South Australia. **E. Harold Brown**,

M.D.Brux., Major I.M.S., appointed Clinical Assistant to the Chelsea Hospital for Women. **J. F. Butler-Hogan**, B.A.R.U.I., M.D.Brux., D.P.H.Camb., appointed Medical Officer of Health to the Tottenham Urban District Council. **Percival Robert Cook**, M.B.Univ.N.Z., appointed Public Vaccinator for the District of Waipara, New Zealand. **William James Cran**, M.B.Univ.N.Z., appointed Public Vaccinator for the District of Brunner, New Zealand. **W. S. Danks**, M.B.Lond., appointed Civil Medical Officer to the South African Field Force. **C. E. Denning**, L.R.C.S., L K.Q C.P.Irel., appointed Certifying Surgeon under the Factory Act for the Epping District of Essex. **Constance Helen Frost**, M.B.N.Z., appointed Assistant Bacteriologist to the Adelaide Hospital, South Australia. **William Gough**, M.B., B.S., B.Sc., L.R.C.P.Lond., M.R.C.S., appointed House Surgeon to the Hospital for Children and Women, Leeds. **Charles Hicks**, L.R.C.P.&S.Edin., L.F.P.S.Glasg., appointed Certifying Surgeon under the Factory Act for the Horsmonden District of Kent. **Frances Ivens**, M.B.Lond., late Clinical and Assistant Pathologist, appointed House Surgeon to the Royal Free Hospital, Gray's Inn Road. **Herbert Charlton Jonas**, M.B., B.S.Lond., M.R.C.S., L.R.C.P., appointed an Honorary Medical Officer to the Barnstaple and North Devon Infirmary. **Lydia Ann Leney**, M.D.Brux., appointed Oculist in the Medical Department of the School Board for London. **Andrew Little**, M.B., M.S.Aberd., appointed Honorary Surgeon to the Eye and Ear Hospital, Bradford. **W. F. Lloyd**, M.B., B.C.Cantab., appointed Assistant Surgeon to the Windsor Royal Infirmary. **Arthur Francis Lynch**, M.B., B.S.Adel., appointed Honorary Pathologist to the Adelaide Hospital, South Australia. **Murdoch Mackenzie**, F.R.C.S.Edin., appointed Port Health Officer for the Port of Westport, New Zealand. **Olive McDougall**, M.B.Lond., appointed House Physician to the Royal Free Hospital, Gray's Inn Road. **J. A. Nixon**, M.B., B.C.Cantab., appointed Surgeon to the s.s. *Johannesburg*. **F. H. Noke**, M.B.Lond., appointed Assistant House Surgeon to the Metropolitan Hospital. **A. L. Ormerod**, M.D.Oxon., D.P.H., appointed Medical Officer of Health of the City of Oxford. **Richard Purnell**, M.D.St.And., appointed Medical Officer of Health of the Borough of Wells. **Alexander B. Ritchie**, M.B., M.S.Edin., appointed Medical Officer to the Manchester School Board. **E. J. Roberts**, M.B.Univ.N.Z., appointed Port Health Officer for the Port of Nelson, New Zealand. **John Ikin Sangster**, M.R.C.S., appointed a Member of the Board of Advice for the School District of the Burra, South Australia. **Bābā M. Singh Sodhi**, M.B., Ch.B.Edin., appointed Senior Assistant Medical Officer to the Portsmouth Asylum. **Stanley Stephens**, L.R.C.P.Lond., M.R.C.S., appointed Medical Officer to the Newton Abbot (Devon) Workhouse. **W. H. Stott**, L.R.C.P.&S.Edin., L.F.P.S.Glasg., appointed Certifying Surgeon under the Factory Act for the Caton District of Lancashire. **E. C. Williams**, M.R.C.S., L.R.C.P.Lond., appointed Surgeon to the P. and O. s.s. *Peninsular*. **M. D. Wood**, M.D.Durh., appointed Second Assistant Medical Officer to the Hayward's Heath Asylum.

**"The Hospital" Institutional Library.**

"Hospitals and Asylums of the World." 4 Vols., with a Portfolio of Plans, complete £12 12s.
"Burdett's Hospitals and Charities, 1902." 5s.
"Cottage Hospitals, General, Fever, and Convalescent." 10s. 6d.
"Hospital Expenditure: The Commissariat." 2s. 6d.
"A Legal Handbook for the Use of Hospital Authorities." 2s. 6d.
"The Cottage Hospital Case Book or Register of Patients." 10s. and 12s. 6d.
"The Furnishing and Appliances of a Cottage Hospital." 6d.
All these are published by the SCIENTIFIC PRESS, Ltd., and may be obtained through any bookseller, or direct from the publishers, 28 and 29 Southampton Street, Strand, London, W.C.

